# Next generation sequencing analysis reveals that the ribonucleases RNase II, RNase R and PNPase affect bacterial motility and biofilm formation in *E. coli*

**DOI:** 10.1186/s12864-015-1237-6

**Published:** 2015-02-14

**Authors:** Vânia Pobre, Cecília M Arraiano

**Affiliations:** Instituto de Tecnologia Química e Biológica António Xavier, Universidade Nova de Lisboa, Apartado 127, 2781-901, Oeiras, Portugal

**Keywords:** Exoribonucleases, RNase II, RNase R, PNPase, Transcriptome, RNA-Seq, Motility, Biofilm formation

## Abstract

**Background:**

The RNA steady-state levels in the cell are a balance between synthesis and degradation rates. Although transcription is important, RNA processing and turnover are also key factors in the regulation of gene expression. In *Escherichia coli* there are three main exoribonucleases (RNase II, RNase R and PNPase) involved in RNA degradation. Although there are many studies about these exoribonucleases not much is known about their global effect in the transcriptome.

**Results:**

In order to study the effects of the exoribonucleases on the transcriptome, we sequenced the total RNA (RNA-Seq) from wild-type cells and from mutants for each of the exoribonucleases (∆*rnb*, ∆*rnr* and ∆*pnp*). We compared each of the mutant transcriptome with the wild-type to determine the global effects of the deletion of each exoribonucleases in exponential phase. We determined that the deletion of RNase II significantly affected 187 transcripts, while deletion of RNase R affects 202 transcripts and deletion of PNPase affected 226 transcripts. Surprisingly, many of the transcripts are actually down-regulated in the exoribonuclease mutants when compared to the wild-type control. The results obtained from the transcriptomic analysis pointed to the fact that these enzymes were changing the expression of genes related with flagellum assembly, motility and biofilm formation. The three exoribonucleases affected some stable RNAs, but PNPase was the main exoribonuclease affecting this class of RNAs. We confirmed by qPCR some fold-change values obtained from the RNA-Seq data, we also observed that all the exoribonuclease mutants were significantly less motile than the wild-type cells. Additionally, RNase II and RNase R mutants were shown to produce more biofilm than the wild-type control while the PNPase mutant did not form biofilms.

**Conclusions:**

In this work we demonstrate how deep sequencing can be used to discover new and relevant functions of the exoribonucleases. We were able to obtain valuable information about the transcripts affected by each of the exoribonucleases and compare the roles of the three enzymes. Our results show that the three exoribonucleases affect cell motility and biofilm formation that are two very important factors for cell survival, especially for pathogenic cells.

**Electronic supplementary material:**

The online version of this article (doi:10.1186/s12864-015-1237-6) contains supplementary material, which is available to authorized users.

## Background

RNA degradation can rapidly control RNA levels and therefore it plays a central role in the cell metabolism. *Escherichia coli* has three 3′-5′exoribonucleases that accomplish most of the RNA degradative activity: RNase II, RNase R and PNPase [1,2]. These exoribonucleases can have different substrates in the cell even though they have some functional overlapping [1].

RNase II is a hydrolytic exoribonuclease that processively degrades RNA in the 3′-5′ direction, is sensitive to secondary structures, it is also known to stall before it reaches a double-stranded region [3,4]. Although RNase II degrading activity is sequence-independent, its favourite substrate is poly(A) tails. RNase II rapidly degrades poly(A) tails, but it halts if it finds secondary structures such as the Rho-independent terminators. The degradation of polyadenylated stretches by RNase II can paradoxically protect some RNAs because the other exoribonucleases (PNPase and RNase R) need a short poly(A) tail as a “toehold” in order to degrade secondary structures [5-10].

RNase R is another 3′-5′ hydrolytic exoribonuclease from the RNase II family of exoribonucleases [11,12]. RNase R can easily degrade highly structured RNAs, but requires a single stranded region in order to be able to bind to the substrates. It was shown to be a key enzyme involved in the degradation of polyadenylated RNA [11,13-15]. RNase R is also a critical enzyme involved in RNA and protein quality control, namely in the degradation of defective tRNAs and rRNAs and is involved in RNA degradation during *trans*-translation [12,14-16]. The activity of RNase R is modulated according to the growth conditions of the cell and is induced under several stress conditions [16,17]. RNase R is a highly unstable protein in exponentially growing cells, but is stabilized in stationary phase and other stress conditions [18]. Most of the RNase R in exponential phase has been shown to be linked with ribosomal proteins [19,20].

In contrast to RNase II and RNase R, PNPase is a 3′-5′ phosphorolytic enzyme. PNPase activity is blocked by double-stranded RNA structures [4], but it can form complexes with other proteins allowing it to degrade through extensive structured RNA [2]. PNPase is not only a degradative enzyme, but is also capable of adding heteropolymeric tails [21,22]. In exponentially growing *E. coli*, more than 90% of the transcripts are polyadenylated and Rho-dependent transcription terminators were suggested to be modified by the polymerase activity of PNPase [23].

Both PNPase and RNase R have also been shown to be involved in virulence in several different organisms [24-27]. In two of these studies PNPase and RNase R were found to affect virulence by altering the motility of the pathogens [25,26]. Motility is extremely important for the cells to survive, especially pathogenic cells that need to colonize different environmental niches [28]. However, under certain conditions, cells can form biofilms that provide several advantages such as antibiotic resistance [29]. Both cell motility and biofilm formation are complex processes and are somewhat correlated, since motile bacteria must become non-motile to form biofilms [30].

The role of exoribonucleases has been extensively studied but there are only two global genomic studies for the exoribonucleases, both done using array technologies and none comparing the three exoribonucleases. In one study Mohanty and Kushner analysed the roles of PNPase and RNase II in mRNA abundance and decay in *E. coli* [31], while in a different report the role of RNase R in the mRNA turnover in *Pseudomonas putida* was studied [32]. In this work we used deep sequencing, more specifically RNA-Seq, to analyse the transcriptomic differences between *E. coli* wild-type cells and deletion mutants of the three main exoribonucleases (∆*rnb*, ∆*rnr* and ∆*pnp*) in exponentially growing cells. This study is the first transcriptomic analysis of the three exoribonucleases and is the first global analysis of RNase R in *E. coli*.

Surprisingly, the transcriptomic analysis revealed that a very high percentage of transcripts are actually down-regulated in the exoribonuclease mutants when compared to the wild-type control. It was also observed that although the exoribonucleases significantly affect many transcripts only 29 transcripts are significantly affected by all three exoribonucleases. In fact, the transcriptome analysis indicated that all three exoribonucleases affected cell motility and biofilm formation. We further demonstrated that RNase II, RNase R and PNPase significantly impaired the motility of the cells. Moreover, we found that RNase II and RNase R mutants formed more biofilms than wild-type cells and conversely, PNPase mutant did not form biofilms.

## Results

### Transcriptome wide analysis

**Figure 1:**
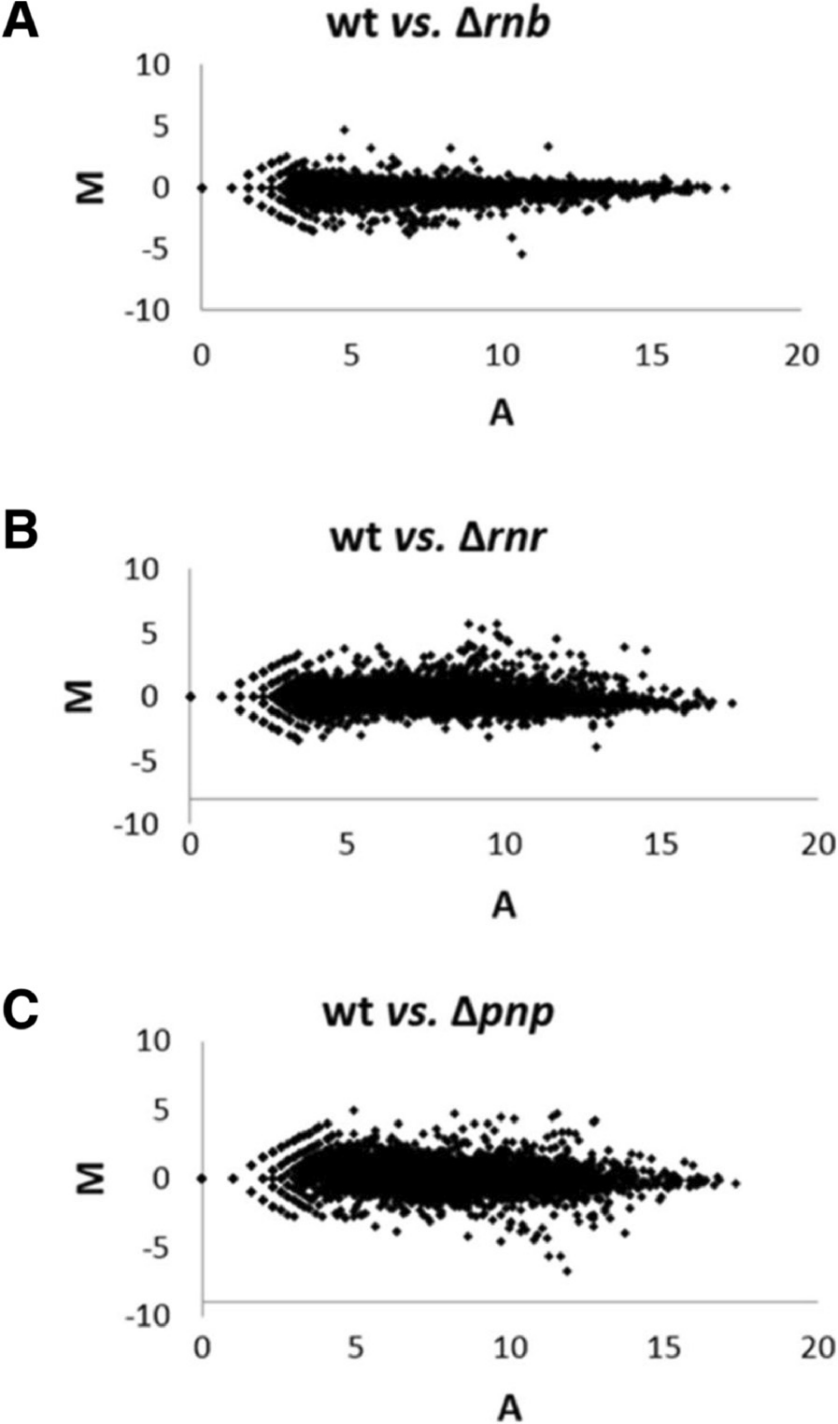
**Transcriptome wide analysis.** Global overview of the transcriptomic differences between the wild-type and the different exoribonucleases mutants. **A)** MA scatterplot comparing wild-type (wt) with ∆*rnb* mutant. **B)** MA scatterplot comparing wild-type (wt) with ∆*rnr* mutant. **C)** MA scatterplot comparing wild-type (wt) with ∆*pnp* mutant. M is the Log2 of the number of reads of the mutant divided by the number of reads of wt, while A is the Log2 of the sum of the two strains. For example, M = log2(∆*rnb*/wt), A = log2(∆*rnb* + wt). Values above 0 correspond to up-regulated transcripts while values below 0 correspond to transcripts down-regulated.

There are three main 3′-5′ exoribonucleases responsible for the degradation of RNA in *E. coli*: RNase II, RNase R and PNPase. In this work we analysed the consequences at the transcriptome level when each of these exoribonucleases were absent from the cell. Therefore we sequenced the total RNA (RNA-Seq) of *E. coli* wild-type cells and of the mutants for each exoribonuclease RNase II (∆*rnb*), RNase R (∆*rnr*) and PNPase (∆*pnp*) growing in exponential phase. The fold-change of all the transcripts was plotted in a MA scatterplot (Figure 1) to obtain an overview of the transcriptomic changes when comparing two samples. Each point in the MA scatterplots corresponds to a transcript. The transcripts with M equal to zero did not change between the two samples that were being compared. On the other hand, transcripts with M above zero are up-regulated while transcripts with M below zero are down-regulated. The dispersion of the log fold-change is not that high for most of the transcripts (Figure 1). However, there were some differences between the different mutants and the wild-type cells. The PNPase mutant is the one that presents higher dispersion of the fold change values (Figure 1C) followed by RNase R mutant (Figure 1B), while the RNase II scatterplot showed low dispersion for most of the transcripts (Figure 1A). This result indicated that PNPase and RNase R had broader effects on gene expression than RNase II. We also calculated the number of transcripts that were up or down-regulated when comparing the different samples (Table 1). The exoribonucleases are involved in the degradation of RNAs, therefore when comparing an exoribonuclease mutant with the wild-type control we would expect to have more up-regulated than down-regulated transcripts. Surprisingly, we found a very high percentage of down-regulated transcripts in all the exoribonucleases mutants when compared to the wild-type control. The percentage of transcripts that were up-regulated when comparing the ∆*rnb* mutant with the wild-type is lower than the percentage of transcripts that were down-regulated (~29% and ~67% respectively). The percentage of down-regulated transcripts was also higher in the ∆*rnr* mutant (~54%). Only PNPase deletion resulted in more up-regulated (~59%) than down-regulated transcripts (~37%), but even in the ∆*pnp* mutant there were still a considerable percentage of down-regulated transcripts (Table 1). The high percentage of down-regulated transcripts in the exoribonuclease mutants might be an indirect consequence of the exoribonuclease deletion, although there is some evidence that some transcripts can be protected instead of being degraded by the exoribonucleases [8,9,31,33]. These results indicate that the role of the exoribonucleases in RNA metabolism is very complex and a deletion of only one of these exoribonucleases can have a great impact in the cell transcriptome.

**Table 1:** **Percentage of up-regulated and down-regulated transcripts**

**Strains**	**% Up-regulated transcripts**	**% Down-regulated transcripts**
**wt** ***vs.*** **Δ** ***rnb***	29.1	66.9
**wt** ***vs.*** **Δ** ***rnr***	41.8	54.5
**wt** ***vs.*** **Δ** ***pnp***	59.0	38.6

### Differential expression analysis of the transcriptome of exoribonucleases mutants

To determine the differentially expressed transcripts, we used the algorithm Cufflinks to calculate the relative abundance of the transcripts. Subsequently, we used the algorithm Cuffdiff to find significant changes in transcript expression, when comparing two samples [34]. We then clustered the list of differentially expressed transcripts into different functional categories using GeneCodis, a web-based tool for the ontological analysis of large lists of genes [35].

**Figure 2:**
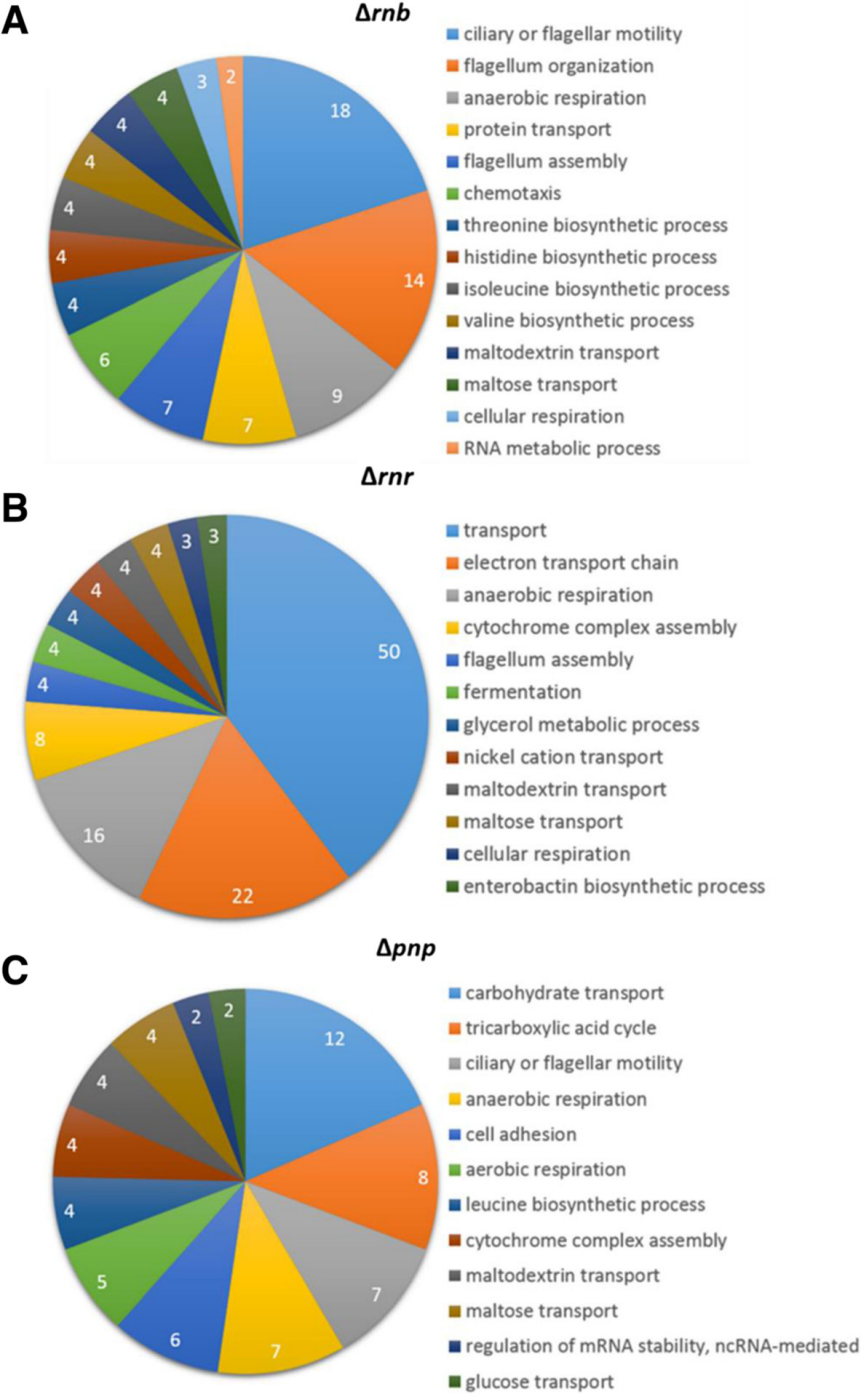
**Differential expression analysis of the transcriptome of the exoribonucleases mutants.** Differentially expressed transcripts distribution in different functional categories. **A)** The differentially expressed transcripts between ∆*rnb* and wild-type. **B)** The differentially expressed transcripts between ∆*rnr* and wild-type. **C)** The differentially expressed transcripts between ∆*pnp* and wild-type. Transcripts were grouped into different functional categories but only the Gene Ontology category of biological process is represented. These results were obtained using GeneCodis [35].

In the RNase II mutant there were 187 transcripts differentially expressed when compared with the wild-type control (Additional file 1: Table S1). Most of the transcripts that were affected by an RNase II deletion were related to flagellum assembly and motility (Figure 2A). Moreover, all the transcripts that were affected by the RNase II deletion and that belong to the Kegg pathway of flagellum assembly were down-regulated (Additional file 1: Table S1). Interestingly, the transcript that was most up-regulated in the ∆*rnb* mutant with a fold change of 10.3 is Antigen-43 (*flu)* known to promote aggregation and inhibit bacterial motility [36]. Therefore, global effects of the RNase II deletion on flagellum assembly can be an indirect effect due to the high levels of antigen-43 in the ∆*rnb* mutant.

The deletion of RNase R affected the expression of 202 transcripts (Additional file 1: Table S2) and most of these transcripts appeared to be involved in transport, anaerobic respiration and electron transport chain (Figure 2B). Similarly to RNase II mutant, RNase R mutant also appeared to affect the expression of transcripts involved in flagellum assembly.

As for the PNPase mutant there were 226 differentially expressed transcripts (Additional file 1: Table S3). Therefore PNPase is the exoribonuclease that significantly affects more transcripts as already anticipated from the MA scatterplots analysis (Figure 1). We clustered these transcripts into functional categories like carbohydrate transport and cellular respiration (Figure 2C). However, even though PNPase affects more transcripts, the number of transcripts grouped into the different functional categories was low, indicating that PNPase affects many different pathways in the cell but does not affect many transcripts of each pathway. A striking difference between ∆*pnp* mutant, ∆*rnb* and ∆*rnr* mutants was the fact that many of the differentially expressed transcripts in ∆*pnp* mutant were stable RNAs (tRNAs, rRNAs and sRNAs). Although in ∆*rnb* and ∆*rnr* these classes of RNAs were also present, they were only a minority. The total number of stable RNAs differentially expressed in ∆*rnb* was 11, in ∆*rnr* was 13 while in the ∆*pnp* there were 53 (Additional file 1: Table S1, S2 and S3). These results were in accordance with other studies that demonstrated that PNPase has a major role in the regulation of small RNAs [33,37,38].

Comparing the ∆*pnp*, ∆*rnb* and ∆*rnr* differentially expressed transcripts, we observed that there was an overlap in the functional categories of the three exoribonucleases (Figure 2). The deletion of any of the exoribonucleases appeared to affect transcripts related to the anaerobic respiration pathway, although deletion of RNase R affected more transcripts involved in anaerobic respiration than deletion of RNase II or PNPase. In the ∆*rnb* and ∆*pnp* mutants the transcripts of the anaerobic respiration were down-regulated in contrast to what happened in the ∆*rnr* mutant (Additional file 1: Tables S1, S2 and S3). Another functional category in which there was an overlap was the flagellum assembly and motility. In all the mutants the differentially expressed transcripts from the flagellum assembly pathway were down-regulated, but deletion of RNase II seemed to have a much higher impact than the deletion of RNase R or PNPase (Additional file 1: Tables S1, S2 and S3). These results suggest that all the exoribonucleases might have an important role in cell motility.

### Overlap between the exoribonucleases

**Figure 3:**
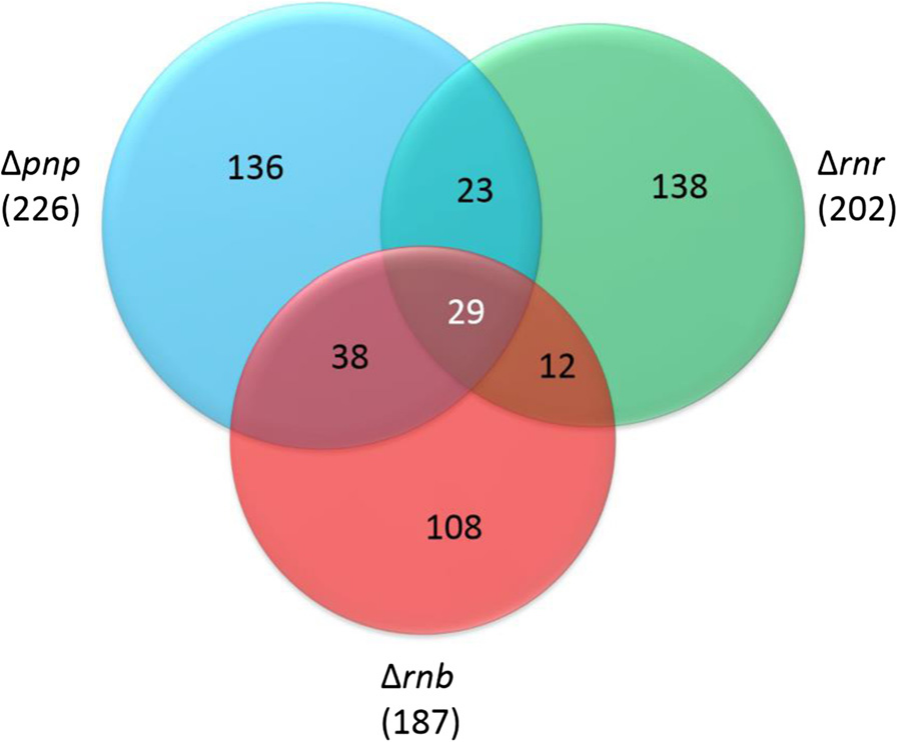
**Overlap between the exoribonucleases.** Venn diagram comparing the number of transcripts that are differentially expressed in each of the three exoribonucleases. A total of 484 transcripts are differentially expressed by the three exoribonucleases. Of those 226 are affected by PNPase, 187 are affected by RNase II and 202 are affected by RNase R. Only 29 transcripts are affected by all exoribonucleases.

Exoribonucleases can show some specificity and even compete among themselves for access to the same RNA substrate. To determine exactly how extensive is the overlap we compared the differentially expressed transcripts from the three exoribonuclease mutants to determine which were affected only by one of the exoribonucleases and those that were affected by more than one exoribonuclease (Figure 3). From the total 484 transcripts that were being differentially expressed by the three exoribonucleases, only 29 transcripts are common to the three exoribonucleases (Figure 3). RNase II and RNase R belong to the same family of enzymes and have very similar catalytic characteristics [11], therefore it was interesting to notice that PNPase shares more transcripts with RNase II (38 transcripts) and RNase R (23 transcripts) than RNase II shares with RNase R (only 12 transcripts). Moreover most of the transcripts that were down-regulated in the ∆*rnb* mutant were up-regulated in the ∆*rnr* mutant. For example, *nir*B (Nitrite reductase [NAD(P)H] large subunit) is down-regulated in ∆*rnb* with a fold-change of 0.36 while in the ∆*rnr* mutant nirB is up-regulated with a fold-change of 9.11 (Additional file 1: Table S1 and S2). The 29 transcripts that were common to the three exoribonucleases are from very distinct functional categories but it appears that most are involved in transport. These results show that although the three exoribonucleases have overlapping roles in the cell, the number of transcripts significantly affected by the three exoribonucleases is not so relevant.

### Deletion of exoribonucleases impairs cell motility

**Figure 4:**
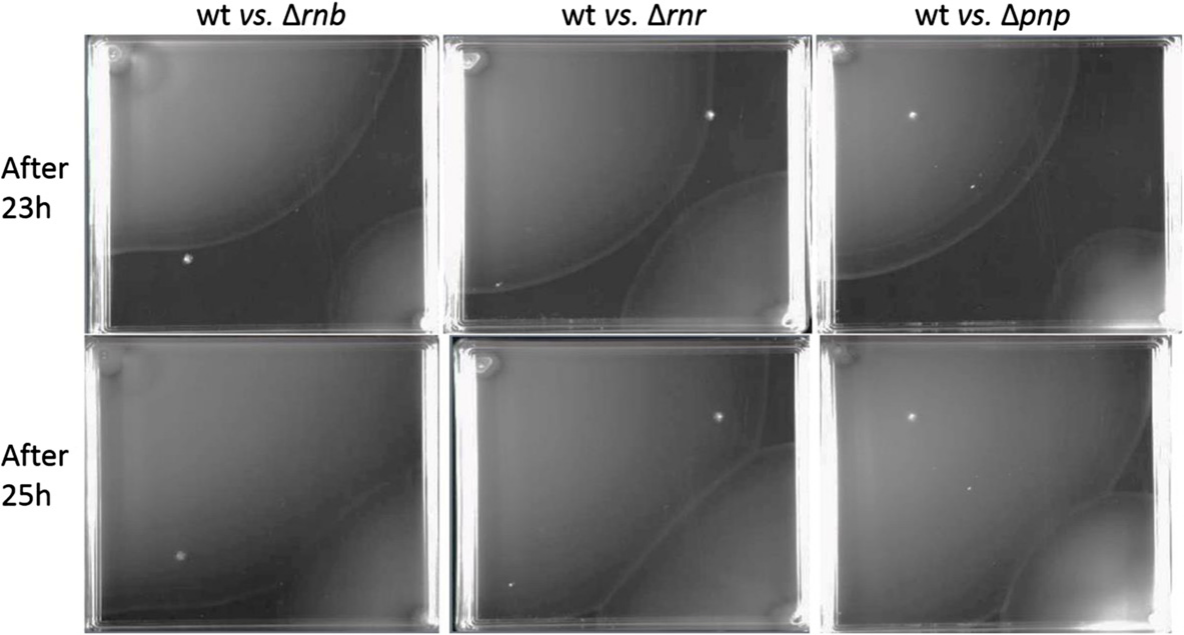
**Deletion of exoribonucleases impairs cell motility.** Swimming ability was assessed on LB agar containing 0.25% agar. Strains were inoculated into the swimming media and incubated at 37°C. Pictures were taken regularly to monitor the increase of the swimming halo, here we are only showing the pictures taken after 23 h and 25 h of inoculation. The upper left corner of the plate was inoculated with wild-type cells and the bottom right corner was inoculated with either ∆*rnb*, ∆*rnr* or ∆*pnp* cells for better comparison between the wild-type and the different mutants.

Our RNA-Seq data suggested that cell motility was significantly affected by the deletion of the exoribonucleases. To verify if in fact the motility of the cells was being affected we performed motility assays and compared the swimming capacity of wild-type cells with the mutants for the different exoribonucleases. We used square plates and inoculated in the same plate the wild-type and one of the mutants. As expected after the RNA-Seq analysis all the exoribonucleases deletion significantly impaired cell motility (Figure 4). Moreover, as suggested by the RNA-Seq data, the RNase R mutant showed a slightly higher swimming ability then the RNase II and PNPase mutants. A previous study had already determined that a *pnp* mutation decreased the motility of the foodborne pathogen *Campylobacter jejuni* [26]. Similarly, the deletion of RNase R in *Aeromonas hydrophila* was reported to reduce these pathogen motility [25]. Both PNPase and RNase R are known to have important roles in virulence of several pathogenic bacteria [1], therefore it was quite interesting that these exoribonucleases affected cell motility since the cell ability to move is of great importance for infection. Interestingly, although RNase II greatly affects cell motility, it has never be found to have any role in virulence. These results prove that RNA-Seq data can be extremely important for finding new roles for the exoribonucleases.

### Exoribonucleases affect biofilm formation

**Figure 5:**
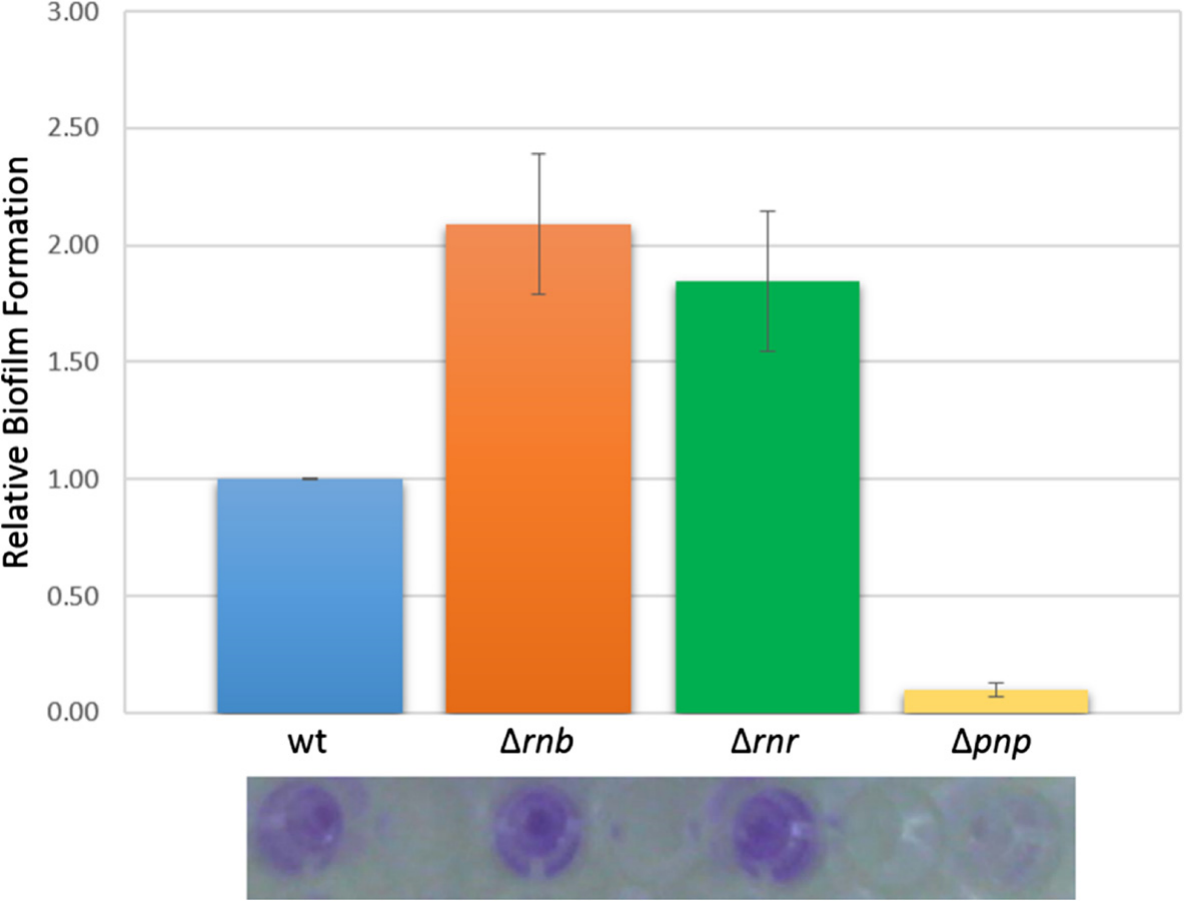
**Exoribonucleases affect biofilm formation.** Effect of the deletion of the exoribonuclease on biofilm production. The different strains (wt, ∆*rnb*, ∆*rnr* and ∆*pnp*) were inoculated into the wells of a fresh microtiter plate and left at 37°C for 24 h. The biofilms formation was measured by determining the OD_550_ after staining with crystal violet. The biofilm formation values were normalized with the OD_600_ of the cultures measured after the 24 h inoculation. The wild-type was used as reference and all other values were obtained by the formula: normalised OD (mutant)/normalised OD (wt). Error bars represent standard deviations.

When analysing more closely the lists of transcripts that were being differentially expressed in the different mutants, we found that both RNase II and PNPase affected antigen-43 expression that, as mentioned previously, is known to promote aggregation of the cells and impair motility [36]. Antigen-43 has also been found to affect biofilm formation in *E. coli* [39]. We also found that there were other biofilm related transcripts being affected by the exoribonuclease deletion besides the antigen-43 (Additional file 1: Tables S1, S2 and S3). Because biofilm formation is inversely correlated with cell motility we hypothesized that the motility impairment could be an indirect effect due to an increase in biofilm formation. We have performed biofilm formation assays to determine if the exoribonucleases mutants did affect the biofilm formation. The RNA-Seq data analysis indicated that RNase II and RNase R mutants were probably able to form more biofilms than the wild-type, and our experimental results confirmed this fact (Figure 5). Surprisingly the PNPase mutant did not formed biofilms. This result was initially unexpected because several transcripts related with biofilm formation were significantly affected in the ∆*pnp* mutant (Additional file 1: Table S3). Similarly ∆*pnp* mutant in *Salmonella* also formed less biofilms then the wild-type control [40]. When analysing more closely our RNA-Seq data we could also find some evidences, corroborating our results for the lack of biofilm formation in the ∆*pnp* mutant. For example, the *bss*R gene that is known to be induced during biofilm formation [41] is significantly down-regulated in the ∆*pnp* mutant. These results show how complex the biofilm formation pathway is and that the RNA-Seq data should be experimentally validated when we are predicting a phenotype.

### RNA-Seq data validation by qPCR

Although we already demonstrated that our RNA-Seq data correctly predicted that the exoribonuclease deletion affected the motility and biofilm formation, we still wanted to validate the fold change values that we obtained by RNA-Seq with quantitative real-time PCR (qPCR). We selected some biofilm and flagellum assembly related transcripts that were differentially expressed in at least one of the exoribonucleases mutant. We then determined the fold change of that transcript by qPCR and compared the values obtained with the RNA-Seq (Table 2). All the values that are above 1 correspond to up-regulated transcripts while the values below 1 correspond to down-regulated transcripts. Even though we are comparing the fold change of the transcripts using two different techniques the results are quite similar. For example, the antigen-43 (Ag43) RNA-Seq fold change for the ∆*rnb*, ∆*rnr* and ∆*pnp* mutants was respectively 10.3, 1.1 and 7.8 while the qPCR fold change for the ∆*rnb*, ∆*rnr* and ∆*pnp* mutants was respectively 12.9, 1.2 and 11.2. These results experimentally validated the RNA-Seq data.

**Table 2:** **Comparison between the values for fold change of some genes using RNA-Seq and qPCR**

**Transcript**	**Mutant**	**RNA-Seq** ^**1**^	**qPCR** ^**1**^
**Ag43 (** ***flu*** **)**	Δ*rnb*	10.3	12.9
	Δ*rnr*	1.1	1.2
	Δ*pnp*	7.8	11.2
**bssR**	Δ*rnb*	0.6	0.6
	Δ*rnr*	5.7	1.6
	Δ*pnp*	0.2	0.4
**flhC**	Δ*rnb*	0.4	0.8
	Δ*rnr*	0.3	0.4
	Δ*pnp*	1.4	2.6
**flgJ**	Δ*rnb*	0.2	0.4
	Δ*rnr*	0.9	0.6
	Δ*pnp*	0.4	0.6
**fliH**	Δ*rnb*	0.2	0.2
	Δ*rnr*	0.8	0.5
	Δ*pnp*	0.3	0.6
**fliA**	Δ*rnb*	0.2	0.2
	Δ*rnr*	0.7	0.6
	Δ*pnp*	0.9	0.8

^1^Fold Changes were calculated as the ratio of mutant to WT. Values above 1 correspond to up-regulated transcripts while values below 1 correspond to down-regulated transcripts.

## Discussion

Our work demonstrated that the deletion of each of the different exoribonucleases has wide-ranging effects on the transcriptome. In this study it was shown that RNase II deletion significantly affected the expression of 187 transcripts while RNase R deletion affected 202 transcripts and PNPase deletion affected 226 transcripts (Additional file 1: Table S1, S2 and S3). Although RNase R is a member of the RNase II family, the two hydrolytic exoribonucleases are very different enzymes. The main difference is that RNase R is able to easily degrade structured RNAs while RNase II activity is blocked by secondary structures [3,4,42]. The differences between these two enzymes are more evident when comparing the transcripts affected by the deletion of RNase II or RNase R (Additional file 1: Table S1 and S2). Of the 389 transcripts affected by RNase II and RNase R only 41 transcripts are affected by both of them (Figure 3). However, most of these transcripts are down-regulated in the ∆*rnb* mutant but up-regulated in the ∆*rnr* mutant. This might indicate that RNase II and RNase R have very distinct roles in the cell. Surprisingly there is a higher overlap in the transcripts affected by PNPase and RNase II or PNPase and RNase R than the overlap between RNase II and RNase R (Figure 3). In fact from the 226 transcripts significantly affected in the PNPase mutant 52 transcripts are also affected in the RNase R mutant and 67 are also affected in the RNase II mutant (Figure 3). These results suggest that PNPase role in the cell overlaps with the role of RNase II and RNase R at a higher extent than the role of RNase II overlaps with the role of RNase R. This is supported by the fact that the double mutant ∆*rnb* ∆*pnp* and ∆*rnr* ∆*pnp* are not viable [16,43,44] while the double mutant ∆*rnb* ∆*rnr* is viable. From the 484 transcripts affected by all exoribonucleases there are only 29 transcripts that are affected in all the exoribonuclease mutants. Although the overlap in the transcripts is not so high the overlap of the functional categories affected by each mutant is more significant (Figure 2).

Interestingly, the deletion of exoribonucleases caused a down-regulation of a high percentage of transcripts (Table 1). This is at first unexpected since the removal of an exoribonuclease should lead to the stabilization and consequently up-regulation of transcripts. Although it has been reported that in some cases an exoribonuclease can protect transcripts from degradation [8,31,33], it is unlikely that all the transcripts down-regulated in the exoribonuclease mutants are a result from such a protection effect. It is plausible that some down-regulated transcripts observed in the exoribonuclease mutants can be due to indirect effect of the deletion of the RNase II, RNase R or PNPase. In 2003, Mohanty and Kushner using microarrays had already described that a high percentage (31%) of *E. coli* mRNAs were decreased in the absence of RNase II [31].

Although many of the up-regulated transcripts can be substrates for the exoribonucleases, it is also possible that some of these transcripts are up-regulated because of an indirect effect of the exoribonucleases. Some transcription factors, for example FliA (σ^28^), are differentially expressed in the exoribonuclease mutants when compared to the wild-type cells (Additional file 1: Table S1, S2 and S3), so transcription could be responsible for some indirect effects of the exoribonucleases in the transcriptome. Moreover, the exoribonucleases and more specifically PNPase can affect the expression of small RNAs [33,37,38] and therefore indirectly affect the expression of their respective targets. Altogether it is important to consider these results as global effects of the exoribonucleases on the cell transcriptome, and not only as direct effects of these enzymes in the transcripts.

All three exoribonucleases affected transcripts from the functional category of flagellum assembly (Figure 2). Most of those transcripts are down-regulated suggesting that the exoribonuclease mutants may present motility deficiencies. In fact, these was what we observed with the motility assays (Figure 4). We have compared our RNA-Seq data with a study of global genomic (microarrays) performed years ago for the RNase II and PNPase mutants [31]. Interestingly, we have observed that most of the flagellum assembly transcripts were also down-regulated [31]. Another study had already showed that the deletion of the RNase R did reduce the motility of the pathogen *Aeromonas hydrophila* [25]. An important transcript that is down-regulated in all three exoribonuclease mutants is the sigma factor fliA. This sigma factor is responsible for initiation of transcription of a number of genes involved in motility and flagella synthesis [45,46]. The down-regulation of this transcript could explain the low motility of the exoribonuclease mutants. Curiously, the transcript which was found to be more up-regulated in the ∆*rnb* mutant with a fold change of 10.3 is antigen-43 (*flu*), a value that was further validated by qPCR (Table 2). Antigen-43 is an autotransporter protein that promotes aggregation, inhibits bacterial motility [36] and has also been linked with biofilm formation [39]. Antigen-43 was also significantly up-regulated in the ∆*pnp* mutant and slightly up-regulated in the ∆*rnr* mutant (Table 2). This led us to the hypothesis that the global effects of exoribonucleases deletion on flagellum assembly could be a consequence of the high levels of antigen-43 which would promote biofilm formation. Additional experiments confirmed that the ∆*rnb* and ∆*rnr* mutants did in fact form more biofilms than the wild-type, but surprisingly the ∆*pnp* mutant did not form more biofilms than the wild-type (Figure 5). This was unexpected because in the PNPase deletion mutant several transcripts implied in biofilm formation, like Antigen-43 and the small RNAs *omr*A and *omr*B [39,47] were affected (Additional file 1: Table S3). A similar result had already been obtained in *Salmonella* were a ∆*pnp* mutant formed less biofilms than the wild-type [40]. Biofilm formation is very complex and there are many genetic alterations during this process [41]. One gene that has been found to be induced during biofilm formation is *bss*R [41] and from our RNA-Seq data was found to be up-regulated in the ∆*rnr* mutant and is significantly down-regulated in the ∆*pnp* mutant (Table 2). We were expecting that *bss*R would also be up-regulated in the ∆*rnb* mutant, however that is not the case. Still there might exist several other factors influencing the formation of biofilms in the absence of the exoribonucleases that need to be more carefully investigated.

In all exoribonuclease mutants there are also several transcripts from the anaerobic respiration functional category which were considerably affected (Figure 2). In fact, the up-regulated transcripts with highest fold-change in the ∆*rnr* mutant can be clustered into this functional category. On the other hand the deletion of RNase II or PNPase leads to a down-regulation of these transcripts (Additional file 1: Table S1, S2 and S3). These result suggests that deletion of RNase II or PNPase can affect the cell respiratory processes but in a different mechanism than RNase R.

Previous studies demonstrated that PNPase and RNase R are involved in the processing and degradation of rRNAs and tRNAs [15,48]. Moreover, Mohanty and Kushner reported that RNase II and PNPase affected the majority of the ribosomal protein mRNAs [31]. In agreement, several of the differentially expressed transcripts in the three mutants were stable RNAs (rRNAs, tRNAs and sRNAs). PNPase is by far the most relevant exoribonuclease affecting 53 of these transcripts while RNase II only affects 11 stable RNAs and RNase R significantly affects 13 stable RNAs. These results demonstrate that PNPase has a very important role in the regulation of these stable RNAs.

## Conclusions

In this work we demonstrate how global transcriptomic analyses can be important to discover new and relevant functions of the exoribonucleases. With the RNA-Seq approach we were able to collect a vast amount of information that considerably expanded our knowledge on the potential targets for the different exoribonucleases in *E. coli*. This work shows that although functional roles of the three exoribonucleases overlap, the number of transcripts affected and the way they are affected can be significantly different. Moreover, this work revealed that deletion of RNase II, RNase R and PNPase decreased the bacterial motility however, while RNase II and RNase R deletions increased the biofilm formation, PNPase deletion was found to significantly impair the cellular ability to form biofilms. These results are also important because arises other questions related to virulence. Motility and biofilm formation are important factors for cell survival and particularly for pathogenic cells. RNase R and PNPase had already been linked to virulence by affecting the motility of pathogenic bacteria [25,26]. Our results show that of all the exoribonucleases RNase II is the enzyme that more significantly affects motility and biofilm formation, therefore we should consider that RNase II might also have an important role in virulence although so far there are no studies associating RNase II with virulence.

## Methods

### Strains and growth conditions

*E. coli* K-12 strain MG1693 and its derivatives used in this work are listed in Table 3. Bacteria were grown at 37°C, with shaking at 200 rpm in Luria-Bertani (LB) medium supplemented with thymine (50 μg ml^−1^). When required, antibiotics were present at the following concentrations: kanamycin, 50 μg ml^−1^; tetracycline, 20 μg ml^−1^; streptomycin/spectinomycin 20 μg ml^−1^.

**Table 3:** **Bacterial strains used in this study**

**Strain**	**Relevant genotype**	**Reference**
**MG1693**	*thyA715*	[49]
**CMA201**	*thyA715* ∆*rnb*	[17]
**HM104**	*thyA715* ∆*rnr*	[17]
**SK10019**	*thyA715* ∆*pnp*	[31]

### Total RNA extraction

Overnight cultures from isolated colonies were diluted in fresh medium to an initial OD_600_ ~ 0.03 and grown to exponential phase (OD_600_ ~ 0.3). RNA was isolated following cell lysis and phenol:chloroform extraction as previously described [38]. After precipitation step in ethanol and 300 mM sodium acetate, RNA was ressuspended in MilliQ-water. The integrity of RNA samples was evaluated by agarose gel electrophoresis. Turbo DNase (Ambion) treatment was used to remove contaminant DNA.

### High-throughput sequencing and data analysis

RNA samples (20 μg) were sent to Vertis Biotechnologie AG, Germany, for library preparation and sequencing of libraries using an Illumina HiSeq platform (single end, 50-bp read length). For the library preparation Vertis Biotechnologie AG depleted the ribosomal RNA molecules from the total RNA preparations using the MICROBExpress Bacterial mRNA Enrichment Kit (Ambion). The rRNA depleted RNAs were then fragmented with RNase III and the 5'PPP structures were removed using RNA 5' Polyphosphatase (Epicentre). Afterwards, the RNA fragments were poly(A)-tailed using poly(A) polymerase and a RNA adapter was ligated to the 5´-phosphate of the RNA fragments. First-strand cDNA synthesis was performed using an oligo(dT)-adapter primer and M-MLV reverse transcriptase. The resulting cDNA was PCR-amplified to about 30 ng/μl using a high fidelity DNA polymerase. After the sequencing of the libraries Vertis Biotechnologie AG bioinformatics department did a preliminary analysis of the high-throughput sequencing results which included the cleaning of the sequences and the mapping of the reads against E. coli genome (NC_000913 downloaded from NCBI genome database). For the cleaning of the sequences Vertis Biotechnologie AG removed low quality and Poly(A) sequences and the adapters were trimmed. We then used the mapped files to run Cufflinks (estimates the relative abundance of the transcripts) and after Cuffdiff to find significant changes in transcript expression when comparing two samples [34]. The transcripts lists resulted from Cuffdiff were then analysed using GeneCodis3, a web-based tool for the ontological analysis of large lists of genes [35].

### Motility assays

To measure bacterial motility we inoculated swimming media plates (triptone 10 g L^−1^, NaCl 5 g L^−1^, thymine 50 μg ml^−1^ and agar 0.25%) with 5 μL of cells in exponential phase. Plates were incubated at 37°C and pictures were regularly taken with Chemidoc Imaging system (BioRad) so as to monitor the increase of the swimming halo size. We used square plates to better compare the swimming of the wild-type cells with the swimming of the different exoribonucleases mutants. The upper left corner of the plate was inoculated with wild-type cells and the bottom right corner was inoculated with either ∆*rnb*, ∆*rnr* or ∆*pnp* cells. Motility assays were done at least three times.

### Biofilm formation assays

Biofilm formation assay were carried on according to the protocol described by Merritt *et al.* [50]. Briefly, diluted cultures in LB without antibiotics (OD_600_ ~ 0.05) were inoculated into the wells of a fresh microtiter plate and left at 37°C for 24 h. OD_600_ of the cultures was measured and subsequently used to normalise the data. Planktonic bacteria was removed from each microtiter dish by briskly shaking the dish out followed by at least two washing steps by submerging the plates in water and then vigorously shake out the liquid. 130 μl of 0.1% crystal violet solution was added to each well and left to stain the biofilms for 30 min at room temperature. The crystal violet was shaken out from the plates and again we performed at least two washing steps as previously described. The plates were allowed to air-dry and then the crystal violet was dissolved by adding 200 μl of an 80% ethanol/20% acetone solution to each stained well and left to incubate for 15 min. The dissolved crystal violet was transferred to a cuvette and each well was washed with another 200 μl of 80% ethanol/20% acetone solution. These 200 μl were added to the previous ones and OD_550_ was measured. The biofilm formation was determined by normalizing the OD_550_ values with the OD_600_ values measured after the 24 h inoculation of the plates. Then the wild-type was used as reference and all other values were obtained by the formula: normalised OD (mutant)/normalised OD (wt). Three independent biofilms formation assays were done each with at least four replicas per sample.

### cDNA synthesis and qPCR

cDNA for quantitative RT-PCR was reverse transcribed from extracted RNA with random hexamers using the QuantiTect® Reverse Transcription Kit (Quiagen). The PCR amplification was performed with a Corbett Rotor Gene RG 3000 real-time PCR system and SensiFAST SYBR No-ROX Kit (Bioline). Oligonucleotides used as primers for qPCR are listed in Table 4. Parameters for qPCR were as follows: 95°C for 2 min, 40 cycles of 95°C for 10 sec, 60°C for 15 sec, 72°C for 20 sec. A negative control (without cDNA) was included to each run. A melting curve was obtained from a first step starting from 60 to 95°C, to control specificities of quantitative PCR reaction for each primer pair. Efficiency of amplifications was determined by running a standard curve with several dilutions of cDNA. Relative copy number was determined using the ∆∆Ct method with *cys*G as the reference gene. qPCR was performed in triplicate with, at least, three templates of RNA extracted from independent cultures.

**Table 4:** **Primers used in this work**

**Primer**	**Sequence (5**′**–3**′**)**
flu-FW	AAGCAGCGGCAGCTATGGATTC
flu-Rev	ACCGGCAACCTCTGTTCTCATC
bssS-FW	GTTGCGTTTGCACTACCAGACC
bssS-Rev	ACCAGAGCGTCTGACCAACTTC
bssR-FW	CAGCGAATCGATCTGCTGAACC
bssR-Rev	TGCCTTCCTCAGTTGCACGTATG
flhC-FW	CGATGCGGTGATCAAAGCCTAC
flhC-Rev	ATGCCAGCAGTGGTCCTTCTTC
flgJ-FW	CTGGGATGCGCAATCACTCAAC
flgJ-Rev	ATATTTGCCGCCGGATCTTCGC
fliH-FW	GCGCTACCTTAAGTTTGCATGGC
fliH-Rev	AGACTTTACAGCCGCCAGGATG
fliA-FW	AGCGTGGAACTTGACGATCTGC
fliA-Rev	TTGTAGGGCGTCATAGCGTTCG
cysG-FW	GACGCTGGTGTTCTATATGGG
cysG-Rev	GGCATTCCGTGTTCAATCAG

### Availability of supporting data

The data discussed in this publication have been deposited in NCBI's Gene Expression Omnibus [51] and are accessible through GEO Series accession number GSE60107. Other supporting data are included as additional files.

## Additional file

Additional file 1: Differentially expressed transcript lists. **Table S1.** Differential expressed transcript list for ∆*rnb* mutant. **Table S2.** Differential expressed transcript list for ∆*rnr* mutant. **Table S3.** Differential expressed transcript list for ∆*pnp* mutant.

## References

[1] Andrade JM, Pobre V, Silva IJ, Domingues S, Arraiano CM (2009). The role of 3'-5' exoribonucleases in RNA degradation. Prog Mol Biol Transl Sci.

[2] Arraiano CM, Andrade JM, Domingues S, Guinote IB, Malecki M, Matos RG (2010). The critical role of RNA processing and degradation in the control of gene expression. FEMS Microbiol Rev.

[3] Cannistraro VJ, Kennell D (1999). The reaction mechanism of ribonuclease II and its interaction with nucleic acid secondary structures. Biochem et Biophys Acta.

[4] Spickler C, Mackie A (2000). Action of RNases II and Polynucleotide Phosphorylase against RNAs containing stem-loops of defined structure. J Bacteriol.

[5] Coburn GA, Mackie GA (1996). Overexpression, purification, and properties of *Escherichia coli* ribonuclease II. J Biol Chem.

[6] Folichon M, Marujo PE, Arluison V, Le Derout J, Pellegrini O, Hajnsdorf E (2005). Fate of mRNA extremities generated by intrinsic termination: detailed analysis of reactions catalyzed by ribonuclease II and poly(A) polymerase. Biochimie.

[7] Hajnsdorf E, Steier O, Coscoy L, Teysset L, Régnier P (1994). Roles of RNase E, RNase II and PNPase in the degradation of the *rpsO* transcripts of *Escherichia coli*: stabilizing function of RNase II and evidence for efficient degradation in an *ams pnp rnb* mutant. EMBO J.

[8] Marujo PE, Hajnsdorf E, Le Derout J, Andrade R, Arraiano CM, Régnier P (2000). RNase II removes the oligo(A) tails that destabilize the *rpsO* mRNA of *Escherichia coli*. RNA.

[9] Mohanty BK, Kushner SR (2000). Polynucleotide phosphorylase, RNase II and RNase E play different roles in the *in vivo* modulation of polyadenylation in *Escherichia coli*. Mol Microbiol.

[10] Pepe CM, Maslesa-Galic S, Simons RW (1994). Decay of the IS10 antisense RNA by 3' exoribonucleases: evidence that RNase II stabilizes RNA-OUT against PNPase attack. Mol Microbiol.

[11] Cheng ZF, Deutscher MP (2002). Purification and characterization of the *Escherichia coli* exoribonuclease RNase R. Comparison RNase II J Biol Chem.

[12] Vincent HA, Deutscher MP (2006). Substrate recognition and catalysis by the exoribonuclease RNase R. J Biol Chem.

[13] Andrade JM, Hajnsdorf E, Régnier P, Arraiano CM (2009). The poly(A)-dependent degradation pathway of *rps*O mRNA is primarily mediated by RNase R. RNA.

[14] Awano N, Rajagopal V, Arbing M, Patel S, Hunt J, Inouye M (2010). *Escherichia coli* RNase R has dual activities, helicase and RNase. J Bacteriol.

[15] Cheng ZF, Deutscher MP (2003). Quality control of ribosomal RNA mediated by polynucleotide phosphorylase and RNase R. Proc Natl Acad Sci U S A.

[16] Cairrão F, Cruz A, Mori H, Arraiano CM (2003). Cold shock induction of RNase R and its role in the maturation of the quality control mediator SsrA/tmRNA. Mol Microbiol.

[17] Andrade JM, Cairrão F, Arraiano CM (2006). RNase R affects gene expression in stationary phase: regulation of *ompA*. Mol Microbiol.

[18] Chen C, Deutscher MP (2010). RNase R is a highly unstable protein regulated by growth phase and stress. RNA.

[19] Liang W, Deutscher MP (2013). Ribosomes regulate the stability and action of RNase R. J Biol Chem.

[20] Malecki M, Bárria C, Arraiano CM (2014). Characterization of the RNase R association with ribosomes. BMC Microbiol.

[21] Mohanty BK, Kushner SR (2000). Polynucleotide phosphorylase functions both as a 3' right-arrow 5' exonuclease and a poly(A) polymerase in *Escherichia coli*. Proc Natl Acad Sci U S A.

[22] Slomovic S, Portnoy V, Yehudai-Resheff S, Bronshtein E, Schuster G (2008). Polynucleotide phosphorylase and the archaeal exosome as poly(A)-polymerases. Biochim Biophys Acta.

[23] Mohanty BK, Kushner SR (2006). The majority of *Escherichia coli* mRNAs undergo post-transcriptional modification in exponentially growing cells. Nucleic Acids Res.

[24] Clements MO, Eriksson S, Thompson A, Lucchini S, Hinton JC, Normark S (2002). Polynucleotide phosphorylase is a global regulator of virulence and persistency in *Salmonella enterica*. Proc Natl Acad Sci U S A.

[25] Erova TE, Kosykh VG, Fadl AA, Sha J, Horneman AJ, Chopra AK (2008). Cold shock exoribonuclease R (*VacB*) is involved in *Aeromonas hydrophila* pathogenesis. J Bacteriol.

[26] Haddad N, Tresse O, Rivoal K, Chevret D, Nonglaton Q, Burns CM, Prévost H, Cappelier JM: Polynucleotide phosphorylase has an impact on cell biology of *Campylobacter jejuni. Front Cell Inf Microbio* 2012: doi:10.3389/fcimb.2012.00030 10.3389/fcimb.2012.00030 PMC3417634 22919622

[27] Tobe T, Sasakawa C, Okada N, Honma Y, Yoshikawa M (1992). *vacB*, a novel chromosomal gene required for expression of virulence genes on the large plasmid of *Shigella flexneri*. J Bacteriol.

[28] Josenhans C, Suerbaum S (2002). The role of motility as a virulence factor in bacteria. Int J Med Microbiol IJMM.

[29] de la Fuente-Nunez C, Reffuveille F, Fernandez L, Hancock RE (2013). Bacterial biofilm development as a multicellular adaptation: antibiotic resistance and new therapeutic strategies. Curr Opin Microbiol.

[30] Guttenplan SB, Kearns DB (2013). Regulation of flagellar motility during biofilm formation. FEMS Microbiol Rev.

[31] Mohanty BK, Kushner SR (2003). Genomic analysis in *Escherichia coli* demonstrates differential roles for polynucleotide phosphorylase and RNase II in mRNA abundance and decay. Mol Microbiol.

[32] Fonseca P, Moreno R, Rojo F (2008). Genomic analysis of the role of RNase R in the turnover of *Pseudomonas putida* mRNAs. J Bacteriol.

[33] De Lay N, Gottesman S (2011). Role of polynucleotide phosphorylase in sRNA function in *Escherichia coli*. RNA.

[34] Trapnell C, Williams BA, Pertea G, Mortazavi A, Kwan G, van Baren MJ (2010). Transcript assembly and quantification by RNA-Seq reveals unannotated transcripts and isoform switching during cell differentiation. Nat Biotechnol.

[35] Tabas Madrid D, Nogales-Cadenas R, Pascual-Montano A (2012). GeneCodis3: a non-redundant and modular enrichment analysis tool for functional genomics. Nucleic Acids Res.

[36] Ulett GC, Webb RI, Schembri MA (2006). Antigen-43-mediated autoaggregation impairs motility in *Escherichia coli*. Microbiology.

[37] Andrade JM, Pobre V, Arraiano CM (2013). Small RNA modules confer different stabilities and interact differently with multiple targets. PLoS One.

[38] Andrade JM, Pobre V, Matos AM, Arraiano CM (2012). The crucial role of PNPase in the degradation of small RNAs that are not associated with Hfq. RNA.

[39] Danese PN, Pratt LA, Dove SL, Kolter R (2000). The outer membrane protein, antigen 43, mediates cell-to-cell interactions within Escherichia coli biofilms. Mol Microbiol.

[40] Saramago M, Domingues S, Viegas SC, Arraiano CM (2014). Biofilm formation and antibiotic resistance in Salmonella Typhimurium are affected by different ribonucleases. J Microbiol Biotechnol.

[41] Schembri MA, Kjaergaard K, Klemm P (2003). Global gene expression in Escherichia coli biofilms. Mol Microbiol.

[42] Frazão C, McVey CE, Amblar M, Barbas A, Vonrhein C, Arraiano CM (2006). Unravelling the dynamics of RNA degradation by ribonuclease II and its RNA-bound complex. Nature.

[43] Deutscher MP (1993). Ribonuclease multiplicity, diversity, and complexity. J Biol Chem.

[44] Donovan WP, Kushner SR (1986). Polynucleotide phosphorylase and ribonuclease II are required for cell viability and mRNA turnover in *Escherichia coli* K-12. Proc Natl Acad Sci USA.

[45] Komeda Y (1986). Transcriptional control of flagellar genes in Escherichia coli K-12. J Bacteriol.

[46] Arnosti DN, Chamberlin MJ (1989). Secondary sigma factor controls transcription of flagellar and chemotaxis genes in Escherichia coli. Proc Natl Acad Sci U S A.

[47] De Lay N, Gottesman S (2012). A complex network of small non-coding RNAs regulate motility in Escherichia coli. Mol Microbiol.

[48] Maes A, Gracia C, Hajnsdorf E, Régnier P (2012). Search for poly(A) polymerase targets in *E. coli* reveals its implication in surveillance of Glu tRNA processing and degradation of stable RNAs. Mol Microbiol.

[49] Arraiano CM, Yancey SD, Kushner SR (1988). Stabilization of discrete mRNA breakdown products in *ams pnp rnb* multiple mutants of *Escherichia coli* K-12. J Bacteriol.

[50] Merritt JH, Kadouri DE, O'Toole GA: Growing and analyzing static biofilms. *Current protocols in microbiology* 2005, Chapter 1:Unit 1B 1. 10.1002/9780471729259.mc01b01s00 PMC4568995 18770545

[51] Edgar R, Domrachev M, Lash AE (2002). Gene Expression Omnibus: NCBI gene expression and hybridization array data repository. Nucleic Acids Res.

